# Protein beverages made of a mixture of egg white and chocolate milk: Microbiology, nutritional and sensory properties

**DOI:** 10.1002/fsn3.983

**Published:** 2019-03-02

**Authors:** Fahimeh Lotfian, Zahra Emam Djomeh, Mostafa Karami, Sohrab Moeini

**Affiliations:** ^1^ Department of Food Science and Technology Islamic Azad University North Tehran Branch Tehran Iran; ^2^ Department of Food Science, Engineering and Technology, Faculty of Agricultural Engineering and Technology University of Tehran Karaj Iran; ^3^ Faculty of Food Science and Technology Bu‐Ali Sina University of Hamedan Hamedan Iran

**Keywords:** chocolate milk, egg white, protein beverage, sensory

## Abstract

Egg white protein and chocolate milk were used for making protein beverage. Microbiological count, sensory attributes, and pH changes were investigated during 10 days of refrigerated storage. At the tenth day, the total numbers of bacteria were between 4.05 and 5.77 (Log CFU/ml). It was concluded that when the amount of egg white increases, the number of bacteria significantly decreases. No coliforms, E. coli, salmonella, mold, and yeast were observed. Also, pH levels were increased from 8.3 to 8.7 and acidic spoilage was reduced. The chocolate milk containing 14% egg white was the most preferred by the panelists in all of the evaluated items except texture. Protein, Ca, Na, and energy contents were increased, and fat, cholesterol, saturated fatty acids, total sugar, lactose, carbohydrate, Mg, Fe, and P contents of the treated samples were decreased.

## 1 INTRODUCTION

Eggs are used in most of the food products and raise the nutritional value and also, improve the functional properties. Ovomucin is one of the egg white proteins that considered a good source of nutrients and have two vital nutrients, protein and carbohydrates. Ovalbumin has the highest amount of egg white protein with good amino acid composition. The rest of the egg white proteins also are considered to be good sources of essential amino acids; thus, egg white can be used as an excellent protein source for many food items (Omana & Wu, 2009). One of the recent developments in the food industry is ready‐to‐drink (RTD) protein beverages with high nutritional value, for muscle building, and increased physical strength. In general, protein beverages are prepared by whey, milk, soy, and egg protein or mixture of them (Dairy Export Council, 2017).

High protein sport nutritional supplements are based on powdered whey proteins, caseins, egg, and soy proteins that are reconstituted with water or milk (Celeghin et al., 2016). Between all of the protein sources, whey is next to egg protein because of its nutritive value. These proteins have unique nutritional and functional properties which can be used for manufacturing protein beverage (Chavan, Shraddha, Kumar, & Nalawade, 2015). In this regard, the most important issues in RTD protein beverages are microbiological properties and pH. Microbial growth is very fast in flavored milk, due to its high water activity and the optimal pH near neutral (6.4‐6.7) and its nutritional properties (Adams & Moss, 2008; Silva et al., 2010; Walkling‐ribeiro, Noci, Cronin, Lyng, & Morgan, 2009). However, dairy proteins generally have a neutral pH and the pH of unspoiled milk is approximately 6.7, a level at which many forms of bacteria thrive (Moseley, 1980). At lower pH levels, lactic acid bacteria can grow and produce lactic acid, then increase the acidity level, and consequently decrease milk quality. Therefore, preventing of reduce pH and increased of bacteria number, are two critical factors to extending the shelf life of these products (Pearson & Marth, 1990). Despite dairy proteins, egg white protein is located in alkaline range. Therefore, it can reduce spoilage. Some researches were done for producing a mixture of milk and the egg. For example, in 2009, shelf life and safety of mixture of liquid whole egg/skim milk with or without cocoa powder were investigated by pulsed electric field (PEF) technology (Pina‐Pérez, Silva‐Angulo, Rodrigo, & Martínez‐López, 2009). With regard to the above descriptions and role of nutritional and functional attributes and health benefits of egg white protein, a new protein beverage with a mixture of egg white and chocolate milk was made and investigated microbiology and its nutritional and sensory properties were assessed. This beverage may be capable to be an innovative product with high energetic and nutritional value to consume for children, elderly, or athletes.

## 2 MATERIALS AND METHODS

### 2.1 Microbiological analysis

The samples were evaluated according to AOAC (2004). The methods were as follows: mesophilic aerobic bacteria (Method 966.23), coliforms (Method 966.23), coliforms and E. coli (Method 991.14), salmonella (Method 996.08), and molds and yeasts (Method 997.07).

### 2.2 Analytical techniques

The samples were analyzed approximately for measuring carbohydrate, total sugar, lactose, fat, protein, and cholesterol content. Percentage of fat was determined by Roese–Gottlieb method at room temperature (AOAC, 2000, 905.02). Protein content was measured by using the Kjeldahl method (AOAC, 1995,999.06). Total sugar and lactose were estimated by Lane and Eynon method as described by (AOAC, 2000, 925.50). Carbohydrate content was calculated by multiplying reducing sugar content by a factor of 0.9. The reducing sugar content was determined using the Fehling's reducing method of Lane and Eynon (AOAC, 2000, 920.18). Cholesterol and fatty acid content of samples were also analyzed by GC method using the AOAC procedure; lipid extract sample was prepared with ethanol/KOH solution at high temperature (75°C). 1 ml of derivative standards and samples were injected into the GC (Hewlett‐Packard Model 5890A). GC was equipped with FID detector and HPCHEM program using Ultra capillary columns. Methylation of fatty acids was done with the method of transesterification (AOAC, 1995, 999.06 and AOAC, 1996a, 994.10). pH of samples was measured at 25°C with pH meter (AZ‐86502 2014, Taiwan) (Institute of Standards and Industrial Research of Iran ,2009). The determination of minerals (Ca, Fe, Mg, Na, Ni, and P) was done with the technique of nitric mineralization and the analysis using spectrophotometry plasma emission (Varian ICP 710, OES, Inductively Coupled Plasma Optical Emission Spectrometers, Palo Alto, CA 94,304‐1,038). The energy value of the product was determined by Microprocessor‐based Automatic Bomb Calorimeter (Singh & Singh, 2012).

### 2.3 Sensory analysis

The sensory evaluation of the samples was conducted by 10 panelists using a score test for flavor, texture, color and appearance, and general acceptability. Samples were tested at a serving temperature of 4°C. The sensory characteristics were assessed on a scale from 1, for very poor, to 5, for excellent (Watts, Ylimaki, Jeffrey, & Elias, 1989).

### 2.4 Preparation of samples

In the present research, a mixed beverage was formulated by pasteurized or sterilized milk (80% v/v) and egg white powder (14 and 16% v/v) (Narin, Hamedan, Iran), cocoa powder (1 and 2% v/v) (Bensdrop, France), sugar (2.8 and 3.8% v/v) (Hafttappeh, Iran), and carrageenan gum (0.05% v/v) (foodINc, China). Therefore, the study was carried out on four beverages: pasteurized milk mixed with 2% cocoa powder +3.8% sugar +14% egg white powder (S1P), pasteurized milk mixed with 1% cocoa powder +2.8% sugar +16% egg white powder (S2P), sterilized milk with 2% cocoa powder +3.8% sugar +14% egg white powder (S1S), and sterilized milk with 1% cocoa powder +2.8% sugar +16% egg white powder (S2S), and 2 control samples: pasteurized milk mixed with 1.3% cocoa powder +3.3% sugar (control p) and sterilized milk with 1.3% cocoa powder +3.3% sugar. Samples were packaged in PET bottles and were kept for one, five, seven, and ten days at 4°C. 0.05% carrageenan was added to all of the above‐mentioned formulations.

### 2.5 Statistical analysis

The experimental design used was as follows: four substrates (S1P, S2P, S1S, and S2S) and 2 control (P, S) samples. Each combination repeated in triplicate. The statistical analysis was perfmed using a three‐way analysis of variance by the general linear model. All of the results were statistically analyzed with SPSS statistical analysis software (SPSS 19, PASW) and Microsoft Excel to show possible differences between means.

## 3 RESULTS AND DISCUSSIONS

### 3.1 Microbial counts and shelf life

**Figure 1:**
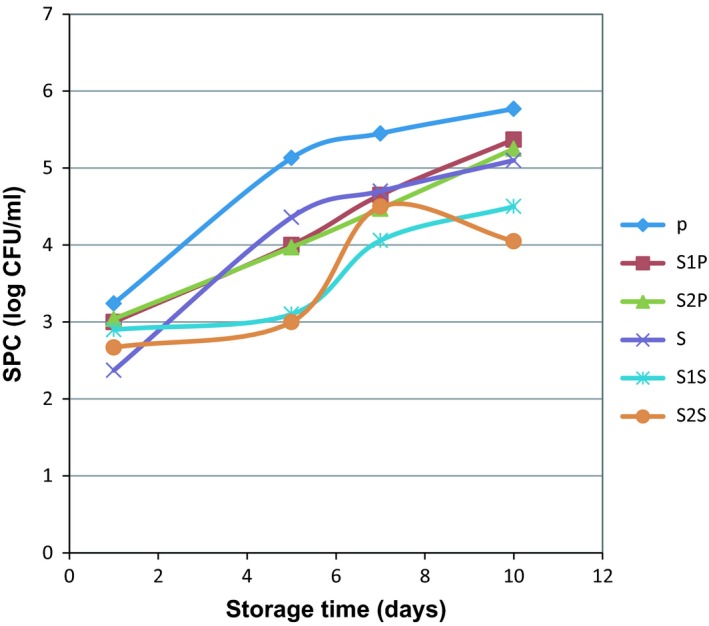
The effect of formulation type and thermal process on SPC

**Figure 2:**
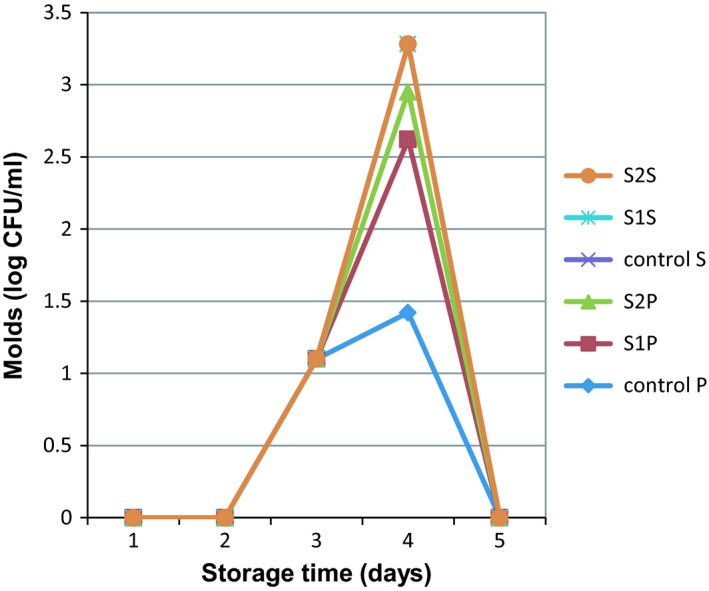
The effect of formulation type and thermal process on the molds number

Standard plate counts of all the samples are shown in Table 1. On the first day, the lowest and highest SPC were for the control S and the control P sample, respectively (2.37 and 4.32 Log10 CFU/ml), which indicates the effect of the thermal process on the number of bacteria. Despite the high SPC in samples containing egg white rather than control samples on the first day, bacterial growth in egg‐free samples (control) was much faster during the storage time. Therefore, on the fifth day, the growth rate increased to 2 logarithmic cycles in the control samples and 0.2‐1 logarithmic cycle in the treated samples (Figure 1). This indicated the effect of egg white addition on preventing the overgrowth of bacteria. Totally, with increasing of the egg white percentage and decreasing percentage of sugar and cocoa powder, the rate of SPC growing, significantly decreased. Finally, the best sample was S2S with 16 percent egg white. Other researchers also proved the effects of sugar and cocoa powder on increasing SPC (Douglas, Gray, Crandall, & Boor, 2000). Therefore, chocolate milk has a short shelf life compared to regular milk (Douglas et al., 2000; Rosenow & Marth, 1987; Woodcock, Hammond, Ralyea, & Boor, 2009). Other studies showed that although milk and chocolate had the same amount of bacteria on the first day, but the number of chocolate milk bacteria was significantly higher than milk at 14th day of storage time. (Douglas et al., 2000). Overgrowth of bacteria can create unpleasant flavors in chocolate milk (Schroder, 1984). Also, until the seventh day, in all samples, except the control sample, no mold and yeast colonies were observed (Table 1). The highest number of molds (1.42 Log10 CFU/ml) was detected in the control samples (P) according to Figure 2. In addition, the results of the other microbial tests such as coliforms, Escherichia coli, and salmonella in all the treated samples and control were negative. Generally, egg white powder is a safe product because it is produced under high drying temperatures; therefore, most of the pathogens, especially salmonella, are killed. For instance, a study showed that SPC of egg white powder, after spray drying, was 51 CFU/g (Baroan et al., 2003). Similar research was reported on the microbiology of protein beverages, for example, whey‐based mango beverage was stored at 4 ± 1 ºC for 30 days. Result showed total viable count (TVC) was high, ranging from 2.60‐2.76 × 10^4^CFU/mL. Yeast and mold count varied between 2.61‐3.60 × 10^3^ CFU/mL, coliforms 3.6‐4.5 × 10^4^ CFU/mL and salmonella were not observed in most of the samples (Ismail, Abdelgader, & Azhari ‐Ali, A., 2011). Analysis of mixed fruit juice with soy milk at 10%, 20%, 30%, 40%, and 50% level without any chemical preservatives showed that bacterial count was higher in SMFJ5 (50:50) (40 x 10^2 ^CFU/ml) and lower in MFJ (100% mixed fruit juice) (11 x 10^2^ CFU/mL). There was no coliform and also molds number was higher juice dilution (Agomuo, Alaka, & Akajiaku, 2015). According to legally definition of Grade A pasteurized milk, standard plate count (SPC) is <20,000 CFU/mL or 4.3 Log10 CFU/mL and <10 coliforms/mL (Food & Drug Administration, 1995). Therefore, according to the results, the S2S sample had the best condition in comparison with all of the other samples on the tenth day of shelf life. As the results showed, egg white was effective on protein content increasing and reduction of bacterial count, which could be due to the presence of some antimicrobial proteins such as lysozyme in egg white. Lysozyme is one of the important proteins in egg white and can control foodborne pathogens (Cegielska‐Radziejewska, Lesnierowski, & Kijowski, 2008). Lysozyme not only has the ability to inhibit the microbial growth, but also has antiviral, anti‐inflammatory, and therapeutic effects (Kovacs‐Nolan, Zhang, Hayakawa, & Mine, 2000). Lysozyme increases the shelf life of food products by inhibiting the growth of microorganisms. Therefore, egg white lysozyme is used as a food preservative. The World Health Organization (WHO) and many countries allow the use of lysozyme as a food preservative (Cunningham, Proctor, & Goetsch, 1991; Mine, Ma, & Lauriau, 2004). It is irrefutable, dry heating has effect on structure of egg, but only primary structures, without any changes in secondary or tertiary structures. On the other hand, chemical modifications of lysozyme after dry heating cause to increase the protein net charge and hydrophobicity of protein in egg white powder (Lechevalier et al., 2017).

**Table 1:** The effect of formulation type and thermal process on total count of bacteria and molds during storage time at refrigerated temperature

Formulation	Thermal process	Total count of bacteria (Log_10 _CFU/ml)				Molds and yeast (Log_10 _CFU/ml)			
		Storage time (day)				Storage time (day)			
		1	5	7	10	1	5	7	10
Control P	Pasteurization	4.32^hi^	5.13^bc^	5.45^ab^	5.77^a^	0.00^a^	0.00^a^	1.10^ab^	1.42^bc^
Control S	Sterilization	2.37^k^	4.36^efg^	4.70^cd^	5.10^bc^	0.00^a^	0.00^a^	0.00^a^	0.33^bc^
S1P	Pasteurization	3^ji^	4.00^fg^	4.65^d^	5.37^ab^	0.00^a^	0.00^a^	0.00^a^	1.20^ab^
S1S	Sterilization	2.90^ji^	3.10^hij^	4.06^efg^	4.50^ed^	0.00^a^	0.00^a^	0.00^a^	0.00^a^
S2P	Pasteurization	3.04^ji^	3.97^g^	4.47^def^	5.25^b^	0.00^a^	0.00^a^	0.00^a^	0.33^bc^
S2S	Sterilization	2.67^jk^	3^ji^	3.55^h^	4.05^efg^	0.00^a^	0.00^a^	0.00^a^	0.00^a^

Note

P = pasteurized, S = sterilized, 1 = formulation 1 (14% egg white), 2 = formulation 2 (16% egg white).

The different letters indicate statistically significant in the column (*p < 0.05*).

### 3.2 Effect of pH on spoilage

**Figure 3:**
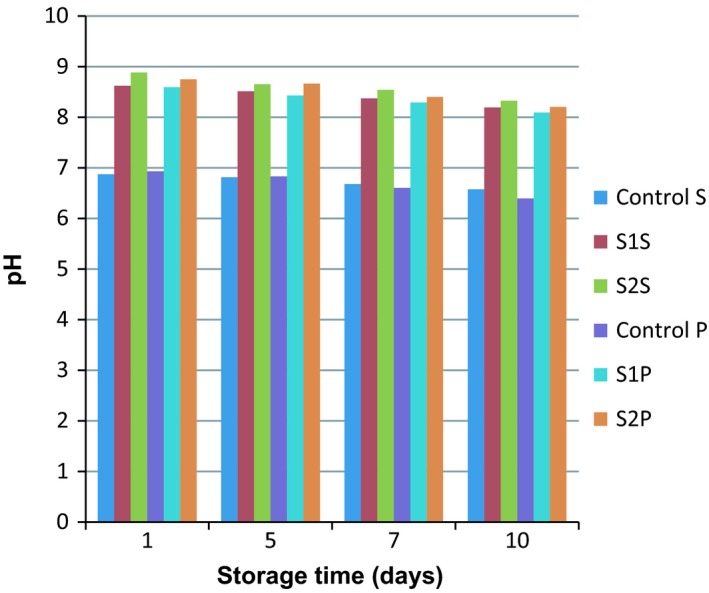
The effects of formulation type and thermal process on pH

pH testing was performed on all of the samples during the shelf life. The results are shown in Table 2. Because egg white is a highly alkaline solution, it was able to increase the pH from 6.8 of the control samples to 8.8 of the treated samples. Also, the effect of the thermal process on the pH of samples was significant and sterilized samples had higher pH rather than the pasteurized ones (Figure 3). The pattern of pH changes during the storage time was descending in all of the samples but after seventh day, pH was below 6.7 in the control samples and higher than 8 in the treated samples. Therefore, it was concluded that egg white can reduce milk spoilage. Moseley (1980) showed that the pH of unspoiled milk was approximately 6.7, a level at which many forms of bacteria thrive. At lower pH levels of 4.0–5.0, lactic acid bacteria can grow and produce lactic acid, then increase the acidity level, and consequently decrease milk quality. Therefore, preventing the reduction in pH and increase of bacteria can extend the shelf life of these products (Pearson & Marth, 1990; Sadhu, 2018). Other studies confirmed the link between pH change and milk spoilage. Fromm and Boor (2004) studied on pasteurized fluid milk during the shelf life and concluded that different bacterial microflora can decrease milk pH. Another research showed that the amount of free fatty acids (FFAs) in the milk increased significantly during the shelf life. Thus, the increase in the amount of FFAs is also related to decrease in pH. Therefore, pH can be used as an index for milk spoilage and milk edibility too (Fromm & Boor, 2004; Lu et al., 2013).

**Table 2:** Interaction of formulation and thermal process on the pH variation of milk samples during storage time at refrigerated temperature

Formulation	Thermal process	Storage time (day)			
		1	5	7	10
Control P	Pasteurization	6.93^k^	6.83^kl^	6.60^m^	6.39^n^
Control s	Sterilization	6.87^kl^	6.81^kl^	6.68^ml^	6.57^mn^
S1P	Pasteurization	8.59^cde^	8.43^efgh^	8.29^hij^	8.09^j^
S1S	Sterilization	8.62^bcd^	8.51^defg^	8.37^ghi^	8.19^ij^
S2P	Pasteurization	8.75^ab^	8.66^bc^	8.40^fghi^	8.20^ij^
S2S	Sterilization	8.88^a^	8.65^bc^	8.54^cdef^	8.32^ghi^

Note

P = pasteurized, S = sterilized, 1 = formulation 1 (14% egg white), 2 = formulation 2 (16% egg white).

The different letters indicate statistically significant in the column (*p < *0.05)*.*

### 3.3 Chemical and mineral characteristics

**Figure 4:**
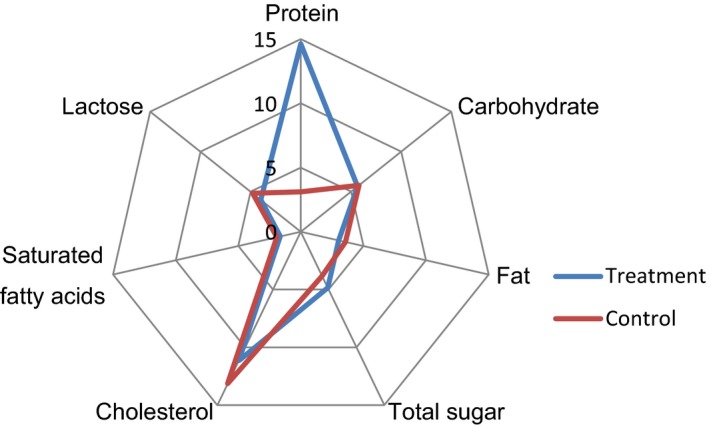
Graphical representation of chemical composition of protein beverage made from egg white and milk compared with the control samples

**Figure 5:**
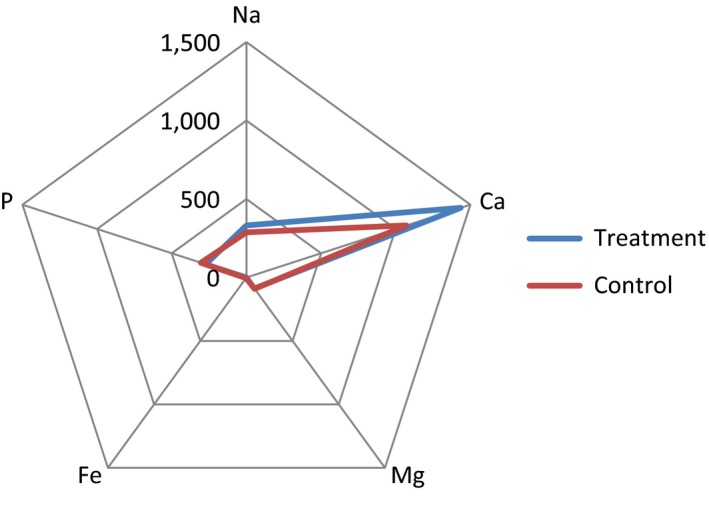
Graphical representation of mineral composition of protein beverage made from egg white and milk compared with the control samples

The results of chemical and mineral analyses are shown in Table 3. Adding EWP had significant effect on the chemical composition of the samples (*p* < 0.05). Increase in EWP increased protein, Ca, Na, and energy contents and also caused a decrease in the fat, cholesterol, saturated fatty acids, total sugar, lactose, carbohydrate, Mg, Fe, and P contents of the treated samples compared to control ones (Figures 4 and 5). Therefore, this protein beverage can be consumed as a low‐fat diet to reduce the risk of cardiovascular disease. The results showed that egg white had a significant effect on the protein content of protein beverage, and the protein content of the samples increased 4.7 times and reached to 14.63%. Egg white has high amount of protein but low amount of calories. In fact, they included around 67% of all the proteins in eggs (Layman & Rodriguez, 2009). Egg white protein is high‐quality and complete food source, because they consist of all nine essential amino acids (Lunven, Le‐Clement‐de‐St‐Marcq, Carnovale, & Fratoni, 1973). Due to high protein content, egg white has some health benefits (Leidy et al., 2015). Traditionally, eggs have been used as the standard of comparison for measuring protein quality because of their essential amino acid (EAA) profile and high digestibility (Layman & Rodriguez, 2009). Egg white is low in cholesterol and fat content; therefore, it is good choice for patients, and for losing weight (Fuller, Sainsbury, Caterson, & Markovic, 2015). Because of valuable nutritional quality with great functional properties, egg white is a substantial ingredient in plenty of food products (Lechevalier et al., 2017). On the other hand, milk and milk products are excellent sources of proteins, lipids, amino acids, vitamins, and minerals. The health benefits of milk cause it to be a good beverage (Haug, Hostmark, & Harstad, 2007). Therefore, when egg white and milk are mixed, it is converted to a unique beverage with nutritional and health benefits and building muscle ability.

**Table 3:** The chemical properties and mineral contents of the treated and control samples

Parameters	Treatment	Control
Protein%	14/63 ± 0/415	3.10 ± 0/10
Fat%	3.02 ± 0/055	3.57 ± 0/0057
Cholesterol%	11.15 ± 0/108	13.14 ± 0/023
Saturated fatty acids%	1.65 ± 0/023	1.92±0.01
Total sugar %	4.48 ± 0/047	3.82 ± 0/04
Carbohydrate %	5.66 ± 0.140	5.79 ± 0/116
Lactose %	4.006 ± 0.030	4.80 ± 0/015
Na (mg/kg)	331.16 ± 1.36	288.76 ± 4.16
Energy (kcal)	125.603 ± 1.22	77.22 ± 0/197
Ca (mg/kg)	1435 ± 2.15	1070.6 ± 2.51
Mg (mg/kg)	86.68 ± 1.25	88.93 ± 1.48
Fe (mg/kg)	3.75 ± 0.091	4.52 ± 0.015
P (mg/kg)	273.93 ± 1.3	303.93 ± 0.0702

### 3.4 Sensory evaluations

**Figure 6:**
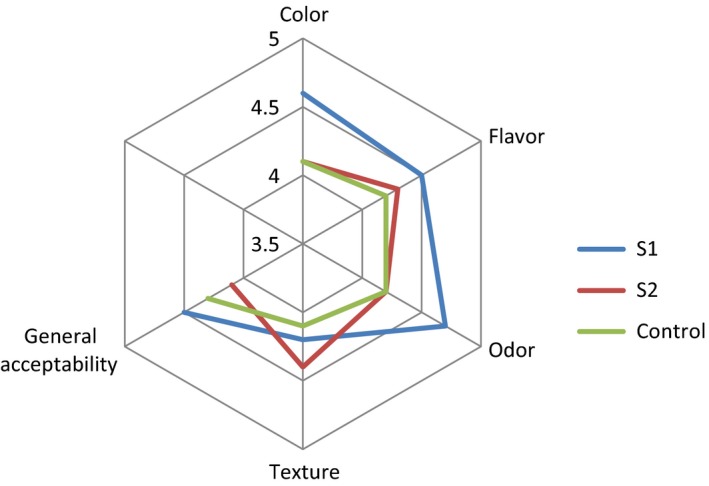
Graphical representation of sensory acceptance of protein beverage made from egg white and milk compared with the control samples

Results of the sensory evaluation of the chocolate milk samples on a scale from 1 (very bad) to 5 (excellent) are shown in a radar plot in Figure 6. Adding 16% EWP had no effect (*p* > 0.05) on the scores of odor and appearance, and flavor. The chocolate milk containing 14% EWP was the most preferred by the panelists in all of the evaluated items except texture. In the same study, sensory evaluation of mixed soy milk drink was investigated and results showed there was no significant difference in taste, aroma, color, mouth feel, and overall acceptability rather than fresh milk (Udeozor, 2012). The study of chocolate dairy beverages with the probiotic bacteria and whey was showed; at maximum concentrations of whey, there were highest sensory attributes for flavor and aroma. Thus, the combination of whey was positive on milk (Silveira et al., 2015). In a mixed drink contained buffalo whey 35%, soy milk 30% and cow milk 35% there were not found any negative changes in the sensory characteristics and its acceptability of the beverage was good during storage period (Macedo, Freitas, Pandey, & Soccol, 1999).

## 4 CONCLUSIONS

In recent decade, consumption of sport foods, especially drinks, had a huge growth. One of the most important of them are protein supplements. Most of the protein supplements are based on whey proteins, caseins, egg, and soy proteins that are reconstituted with water or milk. Between them, because of excellent nutritional qualities, mild flavor, ease of digestibility, and unique functionality and health benefits, egg white and milk protein have been proved. Therefore, this study was investigated on manufacturing new protein beverages made of a mixture of egg white and chocolate milk. An important issue in dairy products is protecting product quality and shelf life and prevention of contamination, but some of the ingredients of chocolate milk, specifically chocolate powder, can increase bacterial counts. Therefore, in this research, no increasing of bacterial number weren't observed. Also, as previously reported, reduction in pH is one of the symptoms of milk spoilage, but egg white in chocolate milk increased pH level and reduced spoilage time of chocolate milk. This phenomenon was related to the presence of some antimicrobial proteins such as lysozyme in added egg white to chocolate milk. Considering the importance of sensory and nutritional properties in protein beverages, this study proved that the addition of egg white to chocolate milk had no significant effect on sensory attributes and led to increase in acceptably. Also, in the case of nutritional characteristics, it was shown that the amount of protein increased and fat, cholesterol, and sugar content decreased; thus, this beverage has beneficial effect on human body and suitable for athletes and children.

## ETHICAL STATEMENT

The study did not involve human and animal testing.

## CONFLICT OF INTEREST

The auths declare that there is no conflict of interests.
